# Impact of Wuyiencin Application on the Soil Microbial Community and Fate of Typical Antibiotic Resistance Genes

**DOI:** 10.1038/s41598-019-40389-w

**Published:** 2019-03-08

**Authors:** Liming Shi, Beibei Ge, Binghua Liu, Xingang Liu, Mingguo Jiang, Kecheng Zhang

**Affiliations:** 1 State Key Laboratory of Biology of Plant Diseases and Insect Pests, Institute of Plant Protection, Chinese Academy of Agricultural Sciences, Beijing, PR China; 2 Guangxi Key Laboratory of Utilization of Microbial and Botanical Resources, Guangxi Key Laboratory for Polysaccharide Materials and Modifications, School of Marine Sciences and Biotechnology, Guangxi University for Nationalities, Nanning, PR China

## Abstract

Antibiotic resistance genes (ARGs) have raised numerous concerns in recent years as emerging environmental contaminants. At present, research on environmental contamination by antibiotics focuses on medical, animal husbandry, and aquaculture fields, with few studies on environmental contamination by agricultural antibiotics in the field of plant protection. Wuyiencin is a low toxicity, high efficiency, and broad-spectrum agricultural antibiotic. It has been widely used in agricultural production and it effectively controls crop fungal diseases. In the present study, pot experiments with four soil treatments (A, B, C and D) were set up in a greenhouse to investigate the effect of the application of wuyiencin on the fate of typical ARGs and microbial community. Eight typical ARGs were detected by real-time PCR and the microbial communities were analyzed using high-throughput sequencing. The results showed that wuyiencin neither significantly influenced ARG abundance and absolute gene copy numbers, nor significantly varied microbial community among treatments. Since it only was short-term results, and the detection number of ARGs was limited, whether wuyiencin is safe or not to ecological environment when using for long-term will need further deep research.

## Introduction

Antibiotics are one of the most important medical discoveries of the 20th century. They have been widely used in the medical field to kill bacteria since their discovery and have played a major role in controlling human infectious diseases and saving countless lives. In addition, antibiotics have been extensively applied in fodder at sub-therapeutic doses for a long time, as well as in aquaculture production worldwide, owing to their disease prevention and growth stimulation properties^1–4^. Moreover, agricultural antibiotics have been extensively applied in plant protection to prevent diseases and control crop pests since the 1940s.

Large-scale antibiotic production and application over more than 70 years has contributed greatly to human health and agricultural production. However, with the proliferation of antibiotic use, evidence has emerged indicating a continuing increase in antibiotic resistance by microorganisms (including pathogenic and non-pathogenic bacteria)^5^. Antibiotic resistance genes (ARGs) enter various pathogens through horizontal gene transfer and pose risks to human health and environmental safety^6^. Since ARGs were first proposed as a novel type of environmental contaminant by Pruden and his colleague, they have attracted considerable attention worldwide^7^. Owing to widespread use of antibiotics in medical and aquaculture fields, environmental pollution has become more severe^8,9^. As reported in 2015, more than two million people per year were infected with antibiotic resistant pathogens, of which 23,000 people dying each year^10^. In addition, numerous studies have demonstrated that the spread of pathogenic bacteria resistance is associated with environmental microbial resistance and ARGs^11,12^. Although many ARGs naturally exist in nature, the widespread use of antibiotics has aggravated the enrichment and transfer of ARGs. Particularly, ARGs in the soil can enter human food chains through absorption by edible plants or leakage into groundwater, which are in turn consumed by humans with potential adverse effects on human health^13^. Although the production, enrichment, and transfer of ARGs have attracted the attention of researchers globally, current research has focused on ARGs in medicine, animal husbandry, and aquaculture^14,15^, with less attention paid to the effects of long-term application of agricultural antibiotics in plant protection on ARGs.

Agricultural antibiotics cover the entire range of pesticides, including insecticides, acaricides, fungicides, viricides, herbicides, and plant growth regulators^16,17^. Avermectins, Jinggangmycin, Kasugamycin, and Polyoxin are typical agricultural antibiotics used extensively. Agricultural antibiotics have numerous advantages. For example, they are safe for humans, animals, and non-target organisms. In addition, they are environmentally friendly, are not easily resisted, but decompose readily. Agricultural antibiotics have been considered as one of the most promising tools for plant-disease control, with numerous potential benefits for nutrition, health, and food production. In recent years, agricultural antibiotics have emerged as a major field in the antibiotics industry, with an increasingly major role in plant-disease control. At present, there are more than 20 kinds of agricultural antibiotics and more than 2000 kinds of dosage forms are used in agricultural production.

Wuyiencin is produced by *Streptomyces ahygroscopicus* var. wuyiensis, which was isolated from a natural soil habitat in Wuyi Mountain, Fujian Province, China, in 1979^18^. It is characterized by low toxicity, high efficiency, and a broad spectrum, in addition to being a pollution-free biological fungicide when compared with other chemical pesticides^19–21^. In the present study, wuyiencin producing bacteria were sequenced by Next-Next-generation sequencing and a bioinformatics analysis carried out. We observed that wuyiencin-producing bacteria could produce numerous secondary metabolites and the genome contained 39 secondary metabolic gene clusters, including nucleosides, polyketones, tetracyclines, sulfonamides, macrolides etc. (unpublished data). The secondary metabolites play key roles in facilitating prevention of infection and disease by wuyiencin.

While medical antibiotics enter water distribution systems through municipal sewage, and veterinary antibiotics are released into farmland via manure application, antibiotics in the aquaculture industry are directly discharged into the rivers and lakes. Although municipal sewage is treated by activated sludge and livestock manure is treated by composting, most of the antibiotics are still not degraded^20,22,23^. Owing to the use of irrigation and utilization of sludge as manure, copious amounts of antibiotics enter farmlands, resulting in the enrichment of antibiotic-resistant bacteria and ARGs in farmland soil.

Sustainable development is critical in agricultural development. Plant protection activities are increasingly adopting biological pesticides that are environmental friendly, have low toxicity, and have a high efficiency. In addition, the use of agricultural antibiotics as biological pesticides is likely to increase in future. Nevertheless, few studies have explored the impacts of agricultural antibiotics on the environment, particularly on environmental microbes, antibiotic resistant bacteria, and ARGs in soil.

The present study simulates the field planting process with tomatoes planted in a greenhouse and wuyiencin sprayed to control plant diseases. Subsequently, seven soil samples were collected at different periods and soil microbial community was analyzed by high-throughput sequencing technology. Eight typical ARGs were detected, including three tetracycline ARGs, three macrolide ARGs, two sulfonamide ARGs, and one integron I gene (Table S1 in Supporting Information). Consequently, we investigated the effects of wuyiencin application on soil microbial community and ARGs, as well as the interactions among them. The results could provide a theoretical basis for the development of novel agricultural antibiotics and their judicious and safe application.

## Materials and Methods

### Experimental design

Pot experiments with four treatments were conducted in a greenhouse with room temperature maintained at 25 °C. Treatment A only contained basal soil; treatment B had wuyiencin sprayed on the basal soil during planting; and both treatment C and treatment D were supplemented with chemical fertilizers containing NaH_4_Cl at 150 mg/kg DW (dry weight), Ca (H_2_PO_4_)_2_ at 150 mg/kg DW, and KCl at 100 mg/kg DW, with treatment C sprayed without wuyiencin during planting and treatment D sprayed with wuyiencin.

The volume of each pot was approximately 7.0 L with a diameter of approximately 30 cm. 7.0 kg of soil was put into each pot. The soils were air dried, crushed, and sieved through a 2 mm mesh before use. Each treatment had triplicates yielding 12 pots. Milli-Q water was used for irrigation throughout the experiments to avoid the introduction of ARGs.

The experiments were conducted for 120 days. Samples were collected on days 0, 10, 20, 40, 60, 90, and 120. Treatments B and D were sprayed with wuyiencin at day 15, 25, 35, 45, 55, 65. The concentration of wuyiencin was 50 ppm, and the amount used in each tomato plant was 1.17 ml, 2.34 ml, 3.51 ml, 4.68 ml, 5.85 ml and 7.02 ml, respectively. Sampling was carried out at a depth of 10–15 cm with three random sites chosen in each pot. The soils were mixed thoroughly and approximately 5 g of soil collected from each pot. Soils from the three pots in each treatment were further mixed to obtain a representative sample. 28 samples were collected. pH, total organic carbon (TOC), and moisture content (MC) in the samples were determined according to the methods^5,24^.

### DNA extraction and microbial community analysis

Total genomic DNA in the 0.5 g soil samples was extracted in triplicate using a FastDNA Spin Kit (MP Biomedical, France) according to manufacturer’s instructions. A mixture of the triplicate DNA extracts was used in further analysis. The concentrations and quality of the extracted genomic DNA were validated and quantified using 0.8% agarose gel electrophoresis and a NanoDrop 2000 spectrophotometer (Thermo Scientific, USA), respectively, and stored at −20 °C for use in subsequent analyses. Microbial community analysis was performed using bacterial identification universal primer pair 515 F/806R^25^. The small-fragment library construction was supported by Sangon Biotech (Shanghai) Co., Ltd. The Illumina MiSeq sequencing system (Illumina, USA) was employed for pair-end sequencing. The pair-end reads were merged by FLASH with a maximum mismatch rate of 0.1 and then filtered using QIIME quality filters. Subsequently, PCR chimeras were removed in UCHIME and the clean sequences were submitted to the NCBI Sequence Read Archive (SRA) under the project number of PRJNA512172. Taxonomic classification of the clean reads in each sample was performed individually using the Ribosomal Database Project classifier (RDP) (http://pyro.cme.msu.edu/) setting the bootstrap cutoff value at 50% of recommended RDP. The operational taxonomic units (OTUs) were analyzed using the ade4 package in R software, and the OTUs with an average abundance value below 0.01% were removed. The remaining OTUs were used for subsequent analyses.

### Quantitative PCR (qPCR)

Eight commonly detected ARGs (*tetG*, *tetM*, *tetX*, *ermB*, *ermF*, *mefA*, *sulI*, *sulII*), an integron I gene (*intI1*), and 16 s rRNA were quantified. The *intI1* was selected for assessing the potential of horizontal gene transfer (HGT), and 16 s rRNA was used to measure biomass. Plasmids carrying the specific genes as standards were employed in constructing the qPCR standard curves. The reaction system and conditions were followed the description by Zhang^24^ and the reactions were conducted using an ABI Real-time PCR system 7500 (ABI, USA). Triplicate reactions (n = 3) were performed for each sample based on a standard curve and a negative control. Detailed information on primers and annealing temperature used in the qPCR are provided in Table S1 and the corresponding gene amplification efficiencies were between 90% and 105%.

### Data analysis

Gene copies indicated the absolute copy numbers present per unit of DW, while the copy number normalized to 16S rRNA was regarded as the abundance. Principal component analysis (PCA) and Procrustes analysis were conducted using Canoco 5.0 (Microcomputer Power, USA). T and Heatmaps were constructed using Heml 1.0^26^.

## Results and Discussion

### Soil chemical and physical properties

All soil chemical and physical properties are displayed in Table S2. Soil pH remained largely the same at different times and in different treatments during the entire experiment, indicating that the wuyiencin and chemical fertilizer application did not affect the soil pH. However, variations in total organic carbon (TOC) and the moisture content (MC) were observed at different times and in different treatments, which were related to soil biomass and the growth status of the tomato plants. On the first day, the MCs of the air-dried soil samples were relatively low. After planting the tomatoes, the MCs increased considerably and the soil microorganisms reproduced rapidly. Organic carbon is the major source of microbial cell components and energy for the soil microbial communities, in addition to being the major by-product of soil microorganism metabolism. Therefore, TOC increased substantially on the 10th day. Afterwards, following tomato growth and the increasing growth, reproduction, and metabolism of microorganisms, TOC was in a dynamic state.

On day 20, 40, 60, and 90, TOC in all treatments reduced significantly, in the order of B > A > D > C. The decreases could have been because of wuyiencin application, which could have prevented disease in plants leading to vigorous growth and higher soil TOC consumption. Alternatively, soil microorganism biomass could have been affected by wuyiencin application via TOC reduction. Considering the frequency of irrigation and the quantity of water used were similar across treatments, the variation in soil moisture content was attributed to soil biomass and the growth status of tomato plants. MC in treatment C and D on days 60 and 90 were higher than in treatment A and B (Table S2). However, on day 120, the MC in treatment A and D was higher than in treatment B and C. In addition, the differences in MC among the four treatments were not significant in the rest of the periods, which could be due to tomato growth and microbial growth and reproduction, with increased water consumption and in turn reduction in soil MC. In addition, chemical fertilizer was added to treatment C and D, which could have reduced biomass and water consumption resulting in high MC.

### Comparison of the fate of ARGs in different treatments

**Figure 1:**
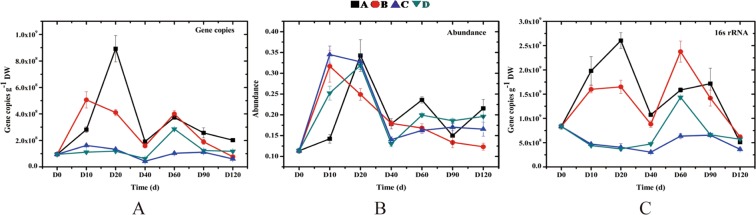
Changes in the ARG abundance, absolute gene copy number, and biomass in soils with four treatments. A, B, C and D represent the four different treatments. Treatment (**A**) only contained basal soil; treatment (**B**) had wuyiencin sprayed on the basal soil during planting; and both treatment (**C**) and treatment D were supplemented with chemical fertilizers containing NaH_4_Cl at 150 mg/kg DW (dry weight), Ca (H_2_PO_4_)_2_ at 150 mg/kg DW, and KCl at 100 mg/kg DW, with treatment C sprayed without wuyiencin during planting and treatment D sprayed with wuyiencin.

During the entire experiment, biomass fluctuated with time (Fig. 1C). In treatment A and B, the microbes grew rapidly following tomato planting from day 0, so that biomass increased rapidly. After day 20, the biomass decreased, potentially due to consumption of nutrients by tomato plants and the interaction with the microbial community. Nevertheless, a decrease in biomass was observed after day 40 followed by another decrease after day 60. There was no significant difference between the final biomass and the initial biomass. However, in treatment C and D, there was an inhibitory effect on microorganism growth due to the application of chemical fertilizers. Therefore, a declining biomass trend was observed. Biomass increased after day 40 but decreased after day 60. There were no significant differences between the final biomass and the initial biomass in treatment C and D.

We also observed that changes in soil pH, TOC, and MC, as well as tomato growth were closely related to biomass, which were the major factors causing fluctuations in biomass. The variations in number of ARGs were consistent with changes in biomass (Fig. 1A). Many studies have reported that biomass succession is a major factor influencing ARG fate, with the succession of ARGs being significantly correlated with changes in biomass^5^. The results of this study are consistent with previous reports. In the present study, the fluctuations in the number of ARGs were mainly attributed to fluctuation in biomass. Due to the homogenization, the abundance trends of the four treatments were generally similar during the entire experiment (Fig. 1B). The results suggest that the chemical fertilizers, as opposed to the wuyiencin, significantly affected biomass; and the usage of wuyiencin significantly influenced neither changes in number of ARGs nor their abundance. Consequently, wuyiencin did not increase the environmental risk of ARGs.

**Figure 2:**
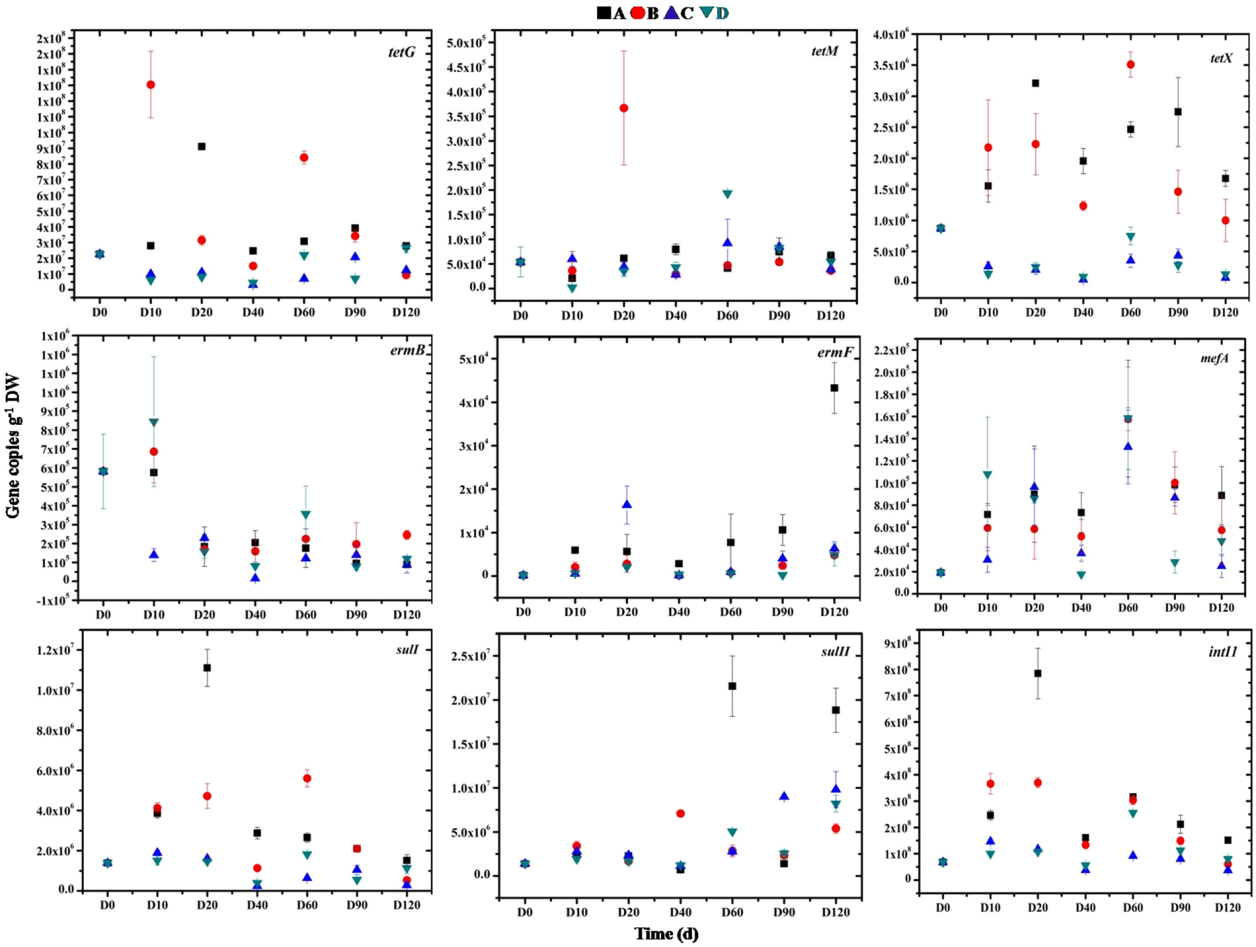
Changes in ARG absolute gene copies in four treatments. A, B, C and D represent the four different treatments. Treatment A only contained basal soil; treatment B had wuyiencin sprayed on the basal soil during planting; and both treatment C and treatment D were supplemented with chemical fertilizers containing NaH_4_Cl at 150 mg/kg DW (dry weight), Ca (H_2_PO_4_)_2_ at 150 mg/kg DW, and KCl at 100 mg/kg DW, with treatment C sprayed without wuyiencin during planting and treatment D sprayed with wuyiencin.

**Figure 3:**
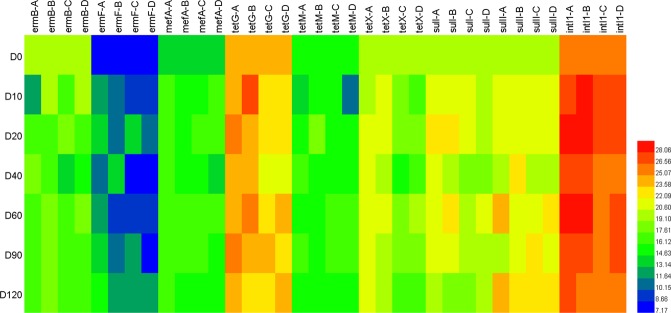
Heatmap comparisons of the absolute gene copies of the target genes (values were log2 transformed). A, B, C, and D represent the four treatments. Treatment A only contained basal soil; treatment B had wuyiencin sprayed on the basal soil during planting; and both treatment C and treatment D were supplemented with chemical fertilizers containing NaH_4_Cl at 150 mg/kg DW (dry weight), Ca (H_2_PO_4_)_2_ at 150 mg/kg DW, and KCl at 100 mg/kg DW, with treatment C sprayed without wuyiencin during planting and treatment D sprayed with wuyiencin.

As shown in Figs 2 and 3, gene copies of *tetG* and *intI1* genes were significantly higher than those of the other seven genes, including *ermB*, *ermF*, *mefA*, *tetM*, *tetX*, *sulI*, and *sulII* in all samples and treatments. *ermB*, *ermF* and *mefA* are macrolide-resistance genes, *sulI* and *sulII* are sulfonamide-resistance genes, while *tetG*, *tetM* and *tetX* represent tetracycline resistance-genes. It was known that wuyiencin-producing strains can produce these metabolites; therefore, genes of macrolide and sulfonamide were selected as the target genes. In addition, tetracycline resistance genes are widely distributed in the environment, and the ARGs are frequently detected in associated studies. Therefore, in the present study, their levels were also quantified. In general, gene copies of all nine genes fluctuated throughout the experiment. However, the final ARG levels did not significantly vary from the initial levels. *ermB* gene copies had increased slightly by day 10, and exhibited a slight decrease after day 10. *ermF* gene copies were the least with a relatively stable level throughout the experiment, except in treatment A where a slight increase was observed. In the other three treatments, the final *ermF* gene copy levels were generally similar to the initial levels. Nevertheless, the *mefA* and *tetX* gene copy levels fluctuated considerably in different treatments at similar periods, in the same treatment at different periods, while the final gene copy levels were not significantly different from the initial levels. In addition, gene copy levels of *tetG*, *tetM*, *sulI* and *sulII* in different treatments were also relatively constant throughout the experiment, only fluctuating at specific time points in some treatments. For example, *tetG* abundance had increased significantly (p < 0.05) by day 10 in treatment B and then fluctuated, but was still not significantly different when compared to the initial level. *tetM* abundance had increased significantly by day 20 in treatment B but decreased steadily with no significant change between the final and initial levels. In treatment A, *sulI* and *sulII* abundance had increased significantly by day 20, by day 60, and by day 120. However, generally, the fluctuation was not significant. *intI1* represents an integron *intI1* gene facilitating the horizontal transfer of ARGs. It is a critical environmental risk factor. It has been demonstrated that *intI1* gene is significantly correlated with ARGs in various environments. The *intI1* gene has also been significantly associated with *tetG*, *sulI* and *sulII* in addition to numerous cases of resistance by microorganisms^9^. In the present study, the *intI1* absolute content and abundance were the highest and there were no significant increases following wuyiencin application. Furthermore, there were no significant correlations with the other eight genes (p > 0.05). The results suggest that the risk of spread to other genes was low, which indicates the environmental safety of wuyiencin application.

### Evolution of microbial community in soils

**Figure 4:**
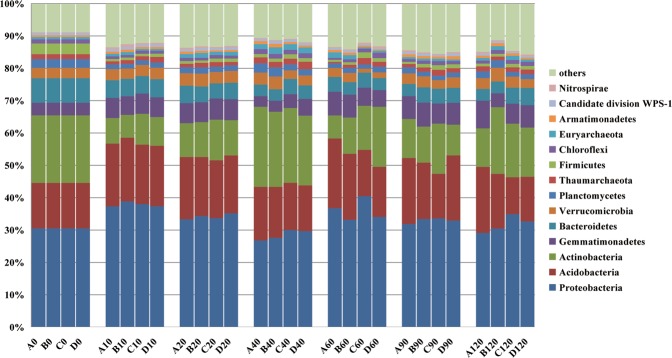
The microbial community of the soils at the Phylum level in different treatments. A, B, C, and D represent the four treatments. Treatment A only contained basal soil; treatment B had wuyiencin sprayed on the basal soil during planting; and both treatment C and treatment D were supplemented with chemical fertilizers containing NaH_4_Cl at 150 mg/kg DW (dry weight), Ca (H_2_PO_4_)_2_ at 150 mg/kg DW, and KCl at 100 mg/kg DW, with treatment C sprayed without wuyiencin during planting and treatment D sprayed with wuyiencin.

The dominant microbes in the four treatments were largely similar; however, there were differences in abundances of the dominant microbes at different times (Fig. 4). The dominant phyla in the soils of four treatments were Proteobacteria (26.81–40.49%), Acidobacteria (11.39–20.55%), and Actinobacteria (7.09–24.75%). Acidobacteria is a taxon in Archaea. During the whole experiment, the abundance of Actinobacteria shifted considerably. The abundance was lowest on day 10 and highest on the day 40. Similarly, Proteobacteria and Acidobacteria abundance fluctuated at different time points but there were no significant differences in abundance among the four treatments at the same time points, suggesting that the application of wuyiencin and chemical fertilizers did not influence Proteobacteria and Acidobacteria abundance and that the changes in abundance could have been due to soil physical and chemical properties during tomato growth. The diversity of microorganisms in the four treatments did not vary significantly at different time points and there were no significant differences in OTU number, Chao1 index, Simpson index and Shannon index (Table S3) indicating that wuyiencin and chemical fertilizer application did not influence both the diversity and abundance of soil microbe communities.

**Figure 5:**
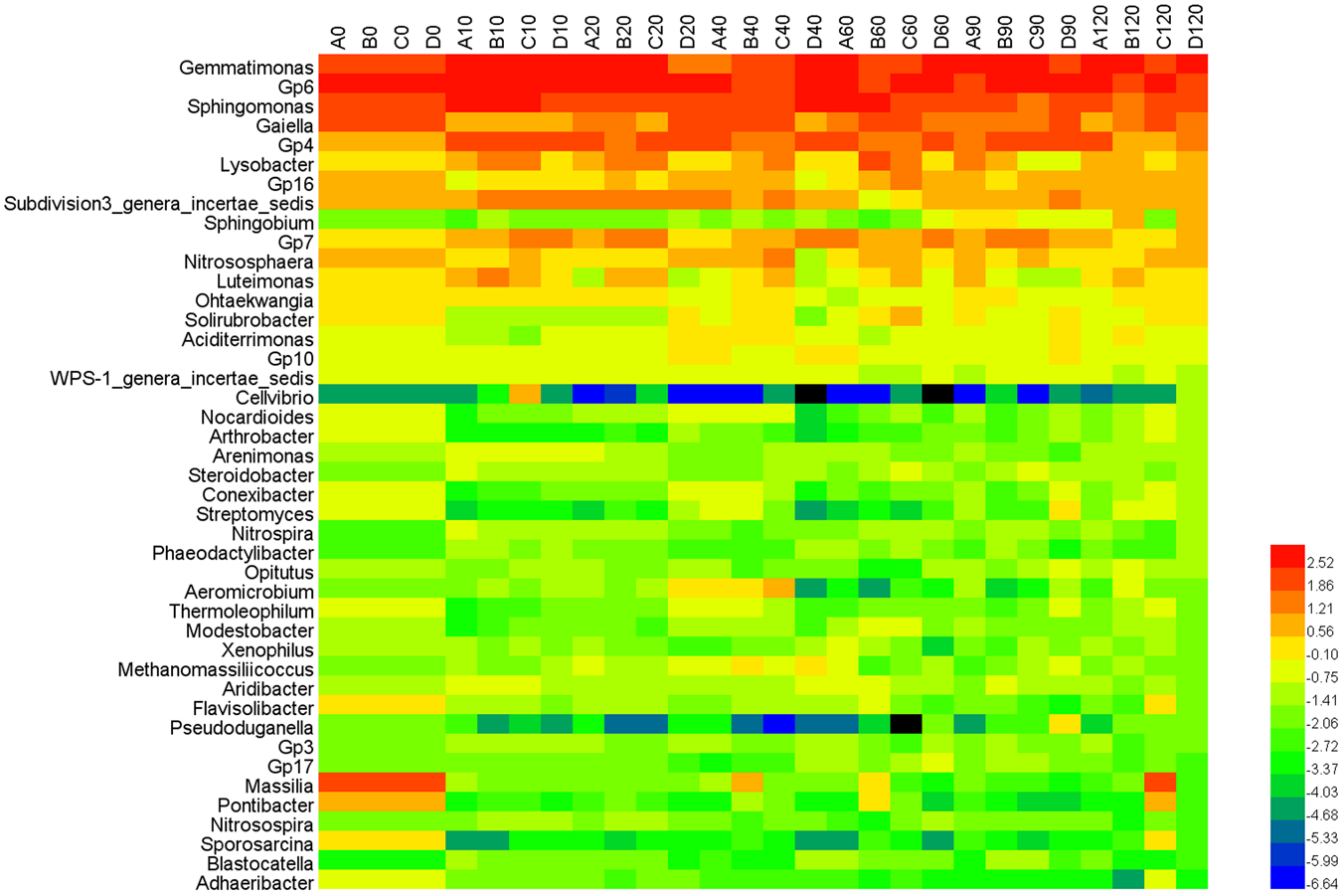
Evolution of the microbial community in soils with different treatments. A, B C, and D represent the four treatments. Treatment A only contained basal soil; treatment B had wuyiencin sprayed on the basal soil during planting; and both treatment C and treatment D were supplemented with chemical fertilizers containing NaH_4_Cl at 150 mg/kg DW (dry weight), Ca (H_2_PO_4_)_2_ at 150 mg/kg DW, and KCl at 100 mg/kg DW, with treatment C sprayed without wuyiencin during planting and treatment D sprayed with wuyiencin.

The dominant genera in samples of the four treatments were *Gemmatimonas* (3.28–8.54%), *Gp6* (3.92–9.02%), *Sphingomonas* (3.05–8.09%), *Gaiella* (1.76–5.32%), and *Gp4* (2.08–4.88%) (Fig. 5). These strains are widely distributed in soil. They have key biological functions and are frequently detected in some related studies. The abundances of the strains fluctuated during the whole experiment but the abundances of the dominant microbes remained relatively similar (Fig. 5) indicating that wuyiencin and chemical fertilizer application had minimal effects on the dominant microbes.

**Figure 6:**
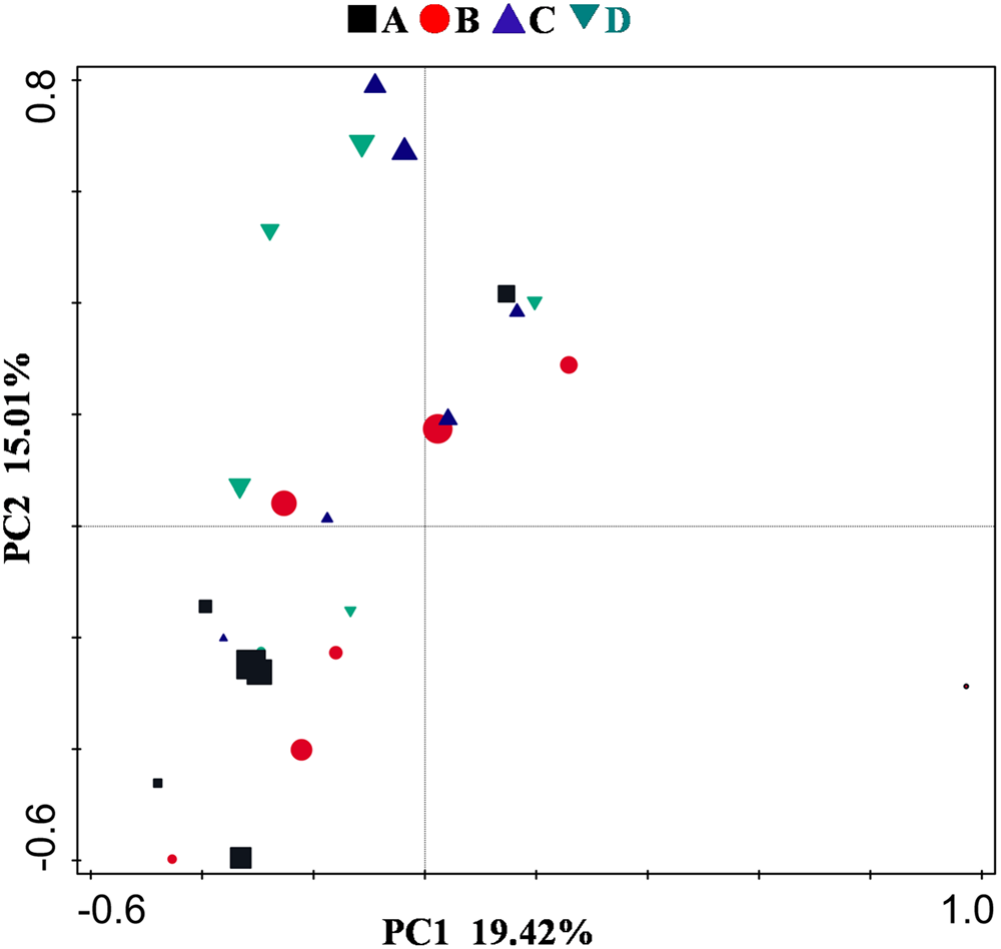
Principal component analysis (PCA) of the evolution of microbial community. Principal component analysis (PCA) based on the OTUs was performed. Increasing symbol size indicates time since tomato growth. A, B C, and D represent the four treatments. Treatment A only contained basal soil; treatment B had wuyiencin sprayed on the basal soil during planting; and both treatment C and treatment D were supplemented with chemical fertilizers containing NaH_4_Cl at 150 mg/kg DW (dry weight), Ca (H_2_PO_4_)_2_ at 150 mg/kg DW, and KCl at 100 mg/kg DW, with treatment C sprayed without wuyiencin during planting and treatment D sprayed with wuyiencin.

According to the results of the PCA analysis (Fig. 6), the addition of wuyiencin and chemical fertilizers resulted in some degree of disturbance within the microbial communities. Nevertheless, wuyiencin application resulted in fewer disturbances in the microbial community, indicating that the microorganisms were not affected by wuyiencin, indicating the safety of wuyiencin in the environment.

All the results showed that soil microbial community changed with tomato growth during the whole experiment. However, there were no significant differences in the changes in dominant microbe abundances. Particularly, microbe abundance did not change substantially before and after the addition of wuyiencin. Soil microbes are a major factor driving soil material transformation. Oxidation, nitrification, ammoniation, nitrogen fixation, and vulcanization activities in soil promote the decomposition of soil organic matter and the conversion of nutrients. Microbes are also critical ARG carriers. The use of wuyiencin did not affect the abundance of soil microorganisms throughout the experiment, which adequately demonstrated that wuyiencin was friendly and safe for the environment.

### Microbial communities influenced ARG fate the most

**Figure 7:**
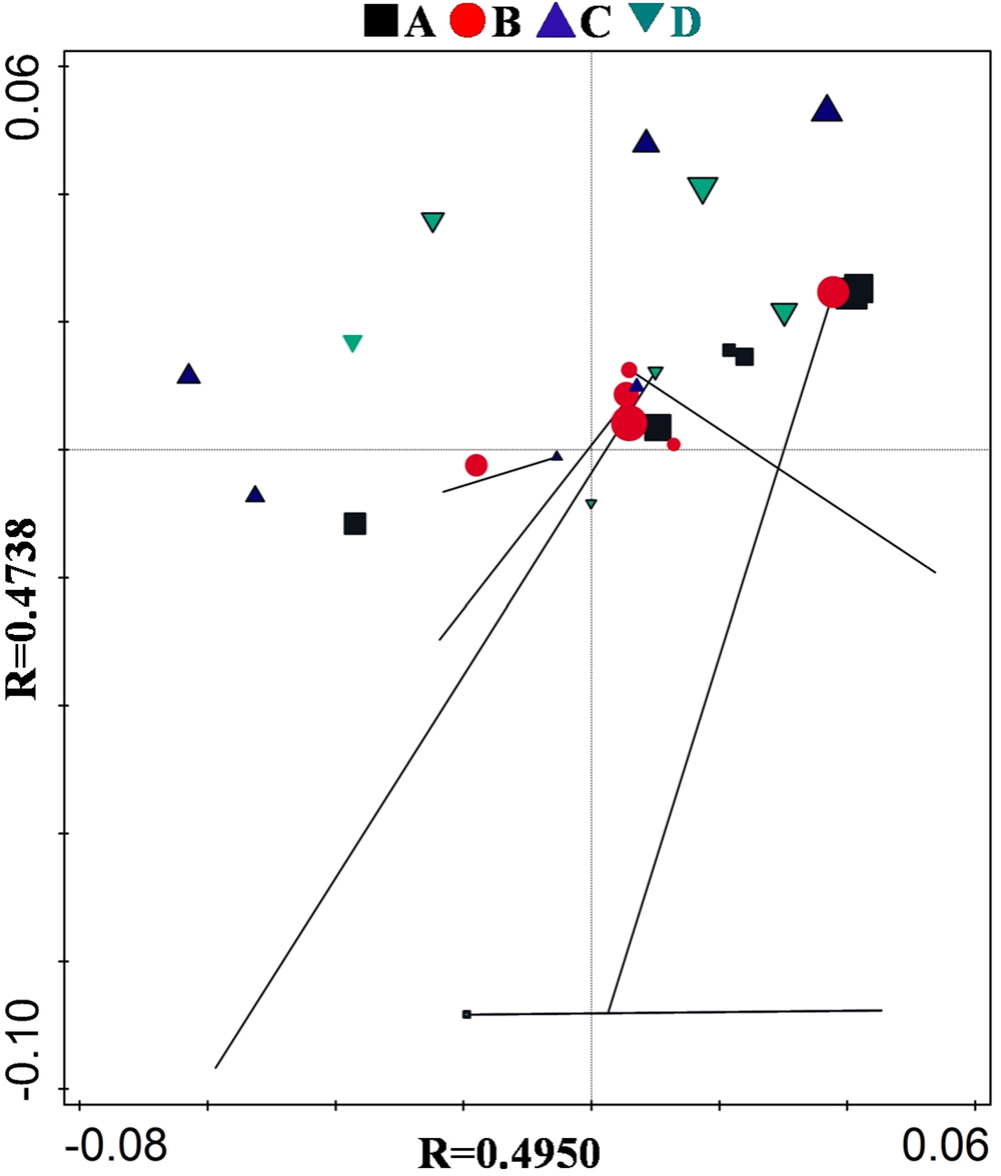
Procrustes analysis based on the evolution of ARGs and microbial community. Increasing symbol size indicated time since tomato growth. A, B C, and D represent the four treatments. Treatment A only contained basal soil; treatment B had wuyiencin sprayed on the basal soil during planting; and both treatment C and treatment D were supplemented with chemical fertilizers containing NaH_4_Cl at 150 mg/kg DW (dry weight), Ca (H_2_PO_4_)_2_ at 150 mg/kg DW, and KCl at 100 mg/kg DW, with treatment C sprayed without wuyiencin during planting and treatment D sprayed with wuyiencin.

ARGs are different from chemical pollutants since their proliferation is through microorganisms. In addition, microbial community can influence the presence or absence of ARGs in different environments, while ARGs require living microorganisms as carriers. Consequently, succession in microbial communities is critical for succession in ARGs. When studying the occurrence of ARGs in the environment, it is essential to investigate relationships between microbial communities and ARGs. To verify the correlation between ARG succession and bacterial community, we carried out Procrustes analysis. The results revealed (Fig. 7) that there was a significant positive correlation between PC1 and PC2 axes with R values of 0.4950 and 0.4738, respectively, indicating that there was a significant positive correlation between ARG succession and bacterial community.

**Figure 8:**
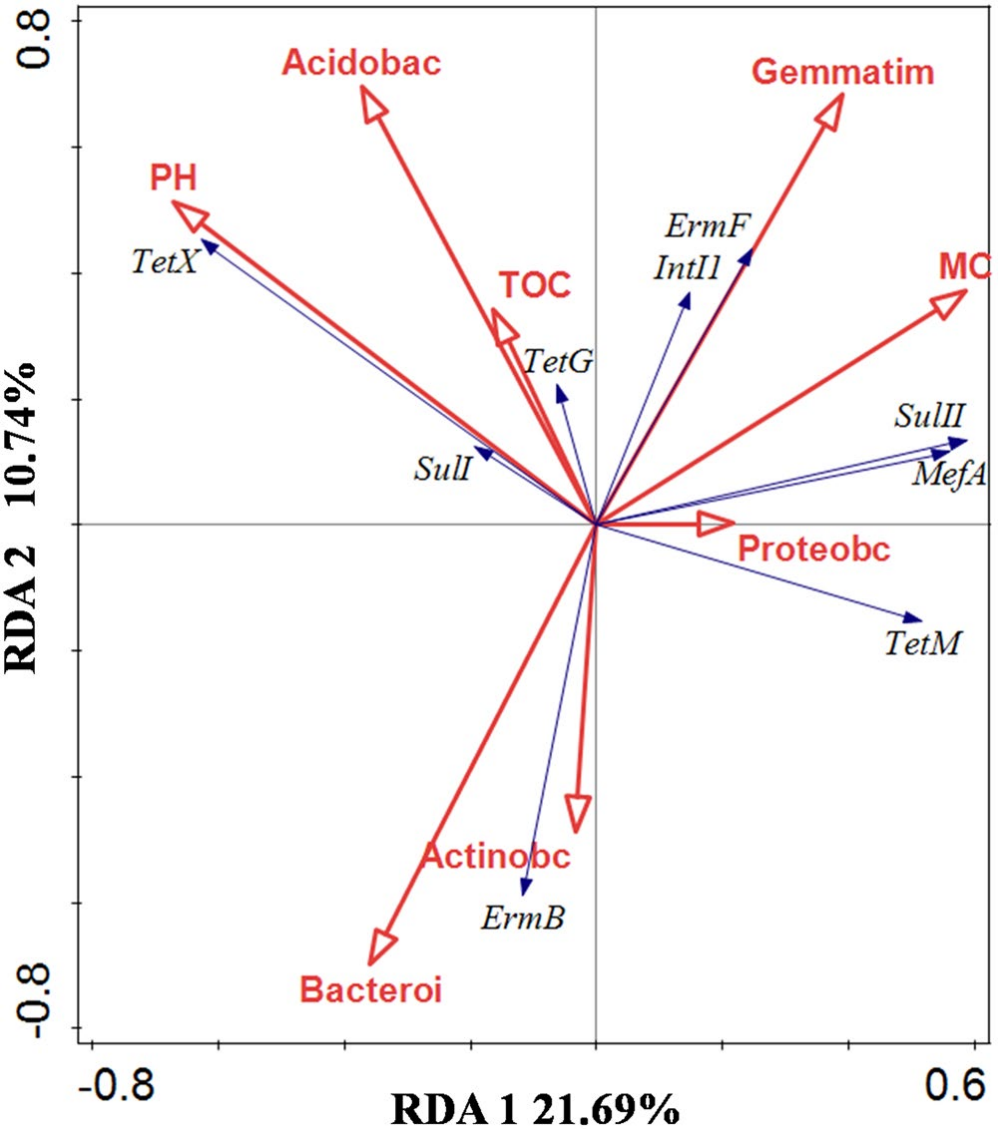
RDA analysis based on OTUs, ARGs and environmental variables. A, B C, and D represent the four treatments. Treatment A only contained basal soil; treatment B had wuyiencin sprayed on the basal soil during planting; and both treatment C and treatment D were supplemented with chemical fertilizers containing NaH_4_Cl at 150 mg/kg DW (dry weight), Ca (H_2_PO_4_)_2_ at 150 mg/kg DW, and KCl at 100 mg/kg DW, with treatment C sprayed without wuyiencin during planting and treatment D sprayed with wuyiencin.

Studies have shown a significant correlation between the horizontal transfer of element *intI1* and some ARGs. In addition, environmental factors can influence changes in ARGs, while there is a significant correlation between bacterial community succession and ARGs present^27,28^. In the present study, at different stages, different factors influence ARG succession, which explains the fluctuations in ARG content and abundance based on a redundancy analysis (RDA). As shown in Fig. 8, *tetX* and *sulI* succession were highly correlated with soil pH, and were somewhat correlated with Acidobacteria. In addition, *tetG* succession was highly correlated with Acidobacteria and TOC, while *ermF* and *intI1* were highly correlated with *Gemmatimonadetes*. *mefA*, *sulII* were highly correlated with Proteobacteria and MC, tetM was highly correlated with Proteobacteria, while *ermB* was highly correlated with Actinobacteria and Bacteroidetes.

Numerous ARGs have a significant correlation with a specific genus, suggesting common distribution characteristics in ARGs. For example, Proteobacteria could be the host flora for *tetM*, *mefA* and *sulII* while Bacteroidetes have been identified as the host flora for *ermB* based on ARDB and CARDB database analyses. In addition, *tetM* is largely present in a variety of horizontal transfer elements, which have broad host populations. *tetM* is likely to enter Proteobacteria through horizontal transfer. In addition, *tetM* has showed significant correlation with the microbial diversity Simpson index, illustrating the wide range of host populations. Furthermore, it was demonstrated that Acidobacteria was the host flora for *tetG*, *tetX* and, *sulI*, and *Gemmatimonadetes* was the host flora for *ermF* and *intI1* (CARD 2017: expansion and model-centric curation of the comprehensive antibiotic resistance database). According to the RDA analysis, there is correlation between bacterial community succession and ARGs.

According to the correlation analysis between bacterial community succession and ARGs, we observed correlations among the horizontal transfer element *intI1*, environmental factors (pH, TOC and MC), bacterial community, and ARG succession. Changes in bacterial community had the greatest impact on ARG succession. Nevertheless, microorganism community was also indirectly affected by the soil physical and chemical properties (pH, TOC and MC) and plant growth. No changes in microbial community were observed following the addition of wuyiencin. In addition, wuyiencin application did not pose ARG risk, the main reasons for this are as follows: first, wuyiencin is sprayed on the plant for prevention fungal diseases, most of them will spray on the plant and only a litter will fall into the soil; second, wuyiencin producing bacteria came from soil microorganisms, that is, wuyiencin can be degraded naturally by soil ecosystems; third, it also can be degraded by environmental factors, such as wind blowing and sun exposure. Consequently, seen from the data showed in this study, wuyiencin application in agricultural production is environmentally friendly,.However, we here just detected eight typical ARGs among more than 2000 reported antibiotics resistance genes, which only preliminary illustrated the effect of wuyiencin to ARGs, presenting extremely limited. Therefore, in subsequently, we will detect more ARGs using high-throughput quantitative PCR method, and conduct a more comprehensive evaluation of the effects of wuyiencin on ARGs. In addition, it only lasted 120 days for this study, and we will investigate the effect of wuyiencin on soil microbial community and ARGs for a longer time in the future. For instance, we will detect the changes of soil microbial community and ARGs when continuously using wuyiencin in the field for 3 to 5 years.

## Conclusions

In conclusion, our results demonstrated the following:

- Biomass present fluctuated during the whole experiment. When wuyiencin was used, the biomass did not change, while the biomass decreased when chemical fertilizer was used.
- The number of antibiotic resistance genes (ARGs) fluctuated during the whole experiment, which was attributed to changes in biomass. There was a significant correlation between ARGs and biomass (p < 0.05). Particularly, when chemical fertilizer was used, the biomass reduced, and in turn, ARG number.
- The abundance of antibiotic resistance genes (ARGs) had did not change due to homogenization. The use of wuyiencin did not significantly affect ARG numbers or abundance. Therefore, wuyiencin did not increase ARG environmental risk.
- Wuyiencin did not cause significant changes in soil microbial community, while microbial community succession was the major driver of ARG fate. In addition, ARG succession was significantly correlated with microbial community. Consequently, in the present study, when wuyiencin was used, microbial community and ARG abundance did not change.

## Supplementary information

Supplemental Figure and Tables
