# Supplementary material for: Impact of Wuyiencin Application on the Soil Microbial Community and Fate of Typical Antibiotic Resistance Genes
Source: Sci Rep. 2019 Mar 8;9:4016. doi: 10.1038/s41598-019-40389-w (PMC6408571; doi:10.1038/s41598-019-40389-w)
Supplement: Supplementary file 1 — Supplemental Figure and Tables [file 41598_2019_40389_MOESM1_ESM.pdf]

Impact of Wuyiencin Application on the Soil Microbial Community and Fate of Typical Antibiotic Resistance Genes

Liming Shi[1], Beibei Ge[1], Binghua Liu[1], Xingang Liu[1], Mingguo Jiang[2], Kecheng Zhang[1*]

1 State Key Laboratory of Biology of Plant Diseases and Insect Pests, Institute of Plant Protection, Chinese Academy of Agricultural Sciences, Beijing, PR China

2 Guangxi Key Laboratory of Utilization of Microbial and Botanical Resources, Guangxi Key Laboratory for Polysaccharide Materials and Modifications, School of Marine Sciences and Biotechnology, Guangxi University for Nationalities, Nanning, PR China

*** Corresponding Author:**

Phone: +86-1062812640; Fax: +86-1062815942

Email: zhangkecheng@sina.com

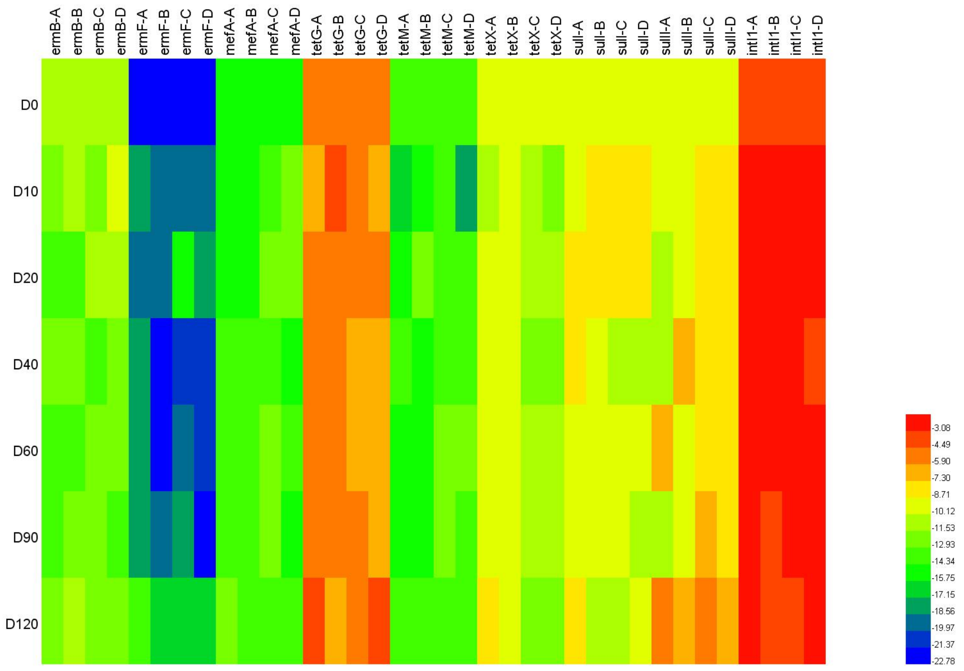

Figure S1: Heatmap comparisons of the abundances of the target genes examined in the presenst study (values were log2 transformed). A, B C, and D represent the four treatments. Treatment A only contained basal soil; treatment B had wuyiencin sprayed on the basal soil during planting; and both treatment C and treatment D were supplemented with chemical fertilizers containing NaH_4Cl at 150 mg/kg DW (dry weight), $Ca(H_2PO_4)_2$ at 150 mg/kg DW, and KCl at 100 mg/kg DW, with treatment C sprayed without wuyiencin during planting and treatment D sprayed with wuyiencin.

Table 1 Primers used in this study

Gene	Primer sequence (5'-3')	Product length (bp)	Annealing temperature (℃)
tetG	GCAGAGCAGGTCGCTGG<br>CCYGCAAGAGAAGCCAGAAG	134	64.2
tetM	ACAGAAAGCTTATTATATAAC<br>TGGCGTGTCTATGATGTTCAC	171	55
tetX	CAATAATTGGTGGTGGACCC<br>TTCTTACCTTGGACATCCCG	468	64.5
ermB	GATACCGTTTACGAAATTGG<br>GAATCGAGACTTGAGTGTGC	364	58
ermF	CGACACAGCTTTGGTTGAAC<br>GGACCTACCTCATAGACAAG	309	56
mefA	TAAGCTTGACATCTCCTCGGCC<br>GCAACGAGGAGATCGAGAAC	348	45
sulI	GTGGACTTTCCGGATTCACC<br>TGAAGCAGAGGGTCTCAAGG	163	55
sulII	GGGAATCGCCTCGTGTGATC<br>ATCCTCCTGCTCCTCTGG	190	60
intI1	CTGGATTTCGATCACGGCACG<br>ACATGCGTGTAAATCATCGTCG	473	60
16s RNA	CGGTGAATACGTTCYCGG<br>GGWTACCTTGTTACGACTT	128	55

Table S2 The physical and chemical properties of soil

Samples	pH	Total organic carbon（g/kg）	Moisture content（%）
A0	8.34±0.01	6.37±0.51	3.42±0.10
B0	8.34±0.01	6.37±0.51	3.42±0.10
C0	8.34±0.01	6.37±0.51	3.42±0.10
D0	8.34±0.01	6.37±0.51	3.42±0.10
A10	8.33±0.11	9.15±0.15	13.78±0.23
B10	8.35±0.03	9.21±0.22	14.73±0.20
C10	7.91±0.02	7.63±0.36	14.00±0.25
D10	7.98±0.03	7.45±0.47	12.26±0.05
A20	8.36±0.03	8.14±0.11	12.00±0.05
B20	8.43±0.11	7.79±0.09	12.59±0.12
C20	7.98±0.03	8.00±0.17	12.05±0.08
D20	8.03±0.02	6.42±0.26	12.78±0.02
A40	8.36±0.04	7.93±0.15	8.07±0.15
B40	8.48±0.01	6.11±0.20	8.53±0.02
C40	8.08±0.04	8.05±0.82	8.63±0.20
D40	8.19±0.01	6.03±0.02	10.34±0.02
A60	8.41±0.01	7.08±0.45	16.60±0.20
B60	8.44±0.15	6.49±0.56	17.94±0.18
C60	8.03±0.01	5.49±0.29	21.60±0.47
D60	8.07±0.13	5.92±0.44	19.77±0.43
A90	8.27±0.21	7.27±0.01	13.47±0.30
B90	8.53±0.02	6.73±0.62	13.57±0.27
C90	8.07±0.17	7.20±0.22	12.93±0.27
D90	8.20±0.08	5.96±0.10	14.49±0.05
A120	8.50±0.01	7.44±0.21	16.84±0.20
B120	8.55±0.03	7.52±0.53	9.72±0.24
C120	8.05±0.21	7.32±0.18	11.92±0.14
D120	8.14±0.01	7.57±0.24	16.44±0.15

Note: A0, A10, A20, A40, A60, A90 and A120 indicate that the samples were collected at days 0, 10, 20, 40, 60, 90, and 120. A, B, C, and D represent the four treatments.

Table S3: The alpha diversity indices of the microbial communities

Sample_ID	Seq_num	OTU_num	Shannon_index	ACE_index	Chao1_index	Coverage	Simpson
A0	67260	5807	6.968727	10026	8435.595	0.966696	0.003973
B0	67260	5807	6.968727	10026	8435.595	0.966696	0.003973
C0	67260	5807	6.968727	10026	8435.595	0.966696	0.003973
D0	67260	5807	6.968727	10026	8435.595	0.966696	0.003973
A10	61346	5856	7.035357	10562.33	8931.281	0.961448	0.003024
B10	48641	5106	6.940498	9119.094	7943.076	0.956600	0.003128
C10	44757	4670	6.850125	8460.257	7173.542	0.956923	0.003700
D10	55311	5245	6.918123	9804.172	8276.173	0.960550	0.003169
A20	53296	5575	7.138684	9813.873	8517.629	0.957877	0.002561
B20	57664	5733	7.145373	10145.79	8503.837	0.960495	0.002397
C20	48457	5096	7.027202	9329.235	7816.644	0.956745	0.002727
D20	52139	5088	6.989726	8854.270	7691.204	0.961142	0.002783
A40	61522	6066	7.197271	8943.895	8999.772	0.961737	0.002676
B40	58626	5802	7.182395	10115.75	8656.410	0.961178	0.002488
C40	56086	5207	7.012821	8989.808	7652.844	0.963699	0.002710
D40	47076	4918	7.025575	8806.389	7563.893	0.957558	0.002612
A60	55853	5050	6.888267	8509.158	7421.883	0.965463	0.003601
B60	67817	5716	7.033819	9553.278	8293.980	0.968548	0.002811
C60	66446	5204	6.847929	7568.922	7483.278	0.970578	0.003322
D60	45510	4709	6.970891	8369.839	7218.500	0.957987	0.002710
A90	50261	5276	7.122849	7785.325	7781.691	0.959193	0.002480
B90	58904	5779	7.179711	8363.029	8303.889	0.962906	0.002374
C90	60103	5329	7.070378	9157.711	7821.703	0.966208	0.002386
D90	55431	5452	7.087694	8093.694	8046.061	0.961466	0.002514
A120	60398	5913	7.179853	8621.870	8558.655	0.962598	0.002390
B120	62502	6052	7.211793	8623.507	8493.340	0.964497	0.002479
C120	56925	5015	7.011862	7091.316	6955.864	0.968327	0.003181
D120	61815	5468	7.104177	9239.648	7932.684	0.966529	0.002314
